# The Complex Interactions Between Obesity, Metabolism and the Brain

**DOI:** 10.3389/fnins.2019.00513

**Published:** 2019-05-24

**Authors:** Romina María Uranga, Jeffrey Neil Keller

**Affiliations:** ^1^Instituto de Investigaciones Bioquímicas de Bahía Blanca, Universidad Nacional del Sur-Consejo Nacional de Investigaciones Científicas y Técnicas, Bahía Blanca, Argentina; ^2^Departamento de Biología, Bioquímica y Farmacia, Universidad Nacional del Sur, Bahía Blanca, Argentina; ^3^Pennington Biomedical Research Center, Louisiana State University System, Baton Rouge, LA, United States

**Keywords:** obesity, overweight, metabolic dysfunction, brain, weight loss

## Abstract

Obesity is increasing at unprecedented levels globally, and the overall impact of obesity on the various organ systems of the body is only beginning to be fully appreciated. Because of the myriad of direct and indirect effects of obesity causing dysfunction of multiple tissues and organs, it is likely that there will be heterogeneity in the presentation of obesity effects in any given population. Taken together, these realities make it increasingly difficult to understand the complex interplay between obesity effects on different organs, including the brain. The focus of this review is to provide a comprehensive view of metabolic disturbances present in obesity, their direct and indirect effects on the different organ systems of the body, and to discuss the interaction of these effects in the context of brain aging and the development of neurodegenerative diseases.

## Obesity

**FIGURE 1:**
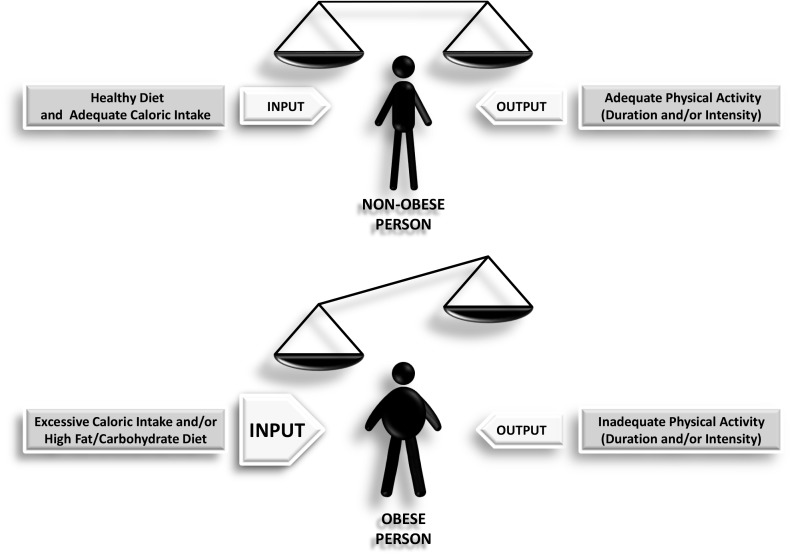
Scheme depicting how the imbalance between the input and the output leads to obesity as a final outcome.

Obesity is often considered to result from excessive calorie consumption (food intake) and/or insufficient or inadequate calorie expenditure (metabolic and physical activity) (Figure 1). Obesity is a complex and chronic non-communicable disease that affects more than a third of the world’s population (Hruby and Hu, 2015). It has been shown that obesity in middle age is able to shorten life expectancy by 4–7 years (Peeters et al., 2003). A major problem with obesity is the diverse set of health associated complications it promotes including hypertension, diabetes, increased cardiovascular risk, and cancer (Calle et al., 2003). The most commonly utilized tool used for measuring obesity today is the body mass index (BMI), defined as a person’s weight in kilograms divided by his or her height in meters squared. By convention, a person with a BMI of less than 25.0 is considered non-obese or “normal,” a person with a BMI between 25.0 and 29.9 is defined as overweight, and a person with a BMI of 30 or more is considered obese. A BMI of more than 40.0 deserves particular attention since it represents morbid obesity (also known as severe or extreme obesity). This index provides a reasonable estimate of body fat, and it is more accurate than skinfold measurements. However, the use of BMI has certain limitations, because it does not distinguish between lean and fat mass, nor does it indicate anything about fat distribution. In this sense, computed tomography or magnetic resonance imaging are the most accurate methods to measure the amount of visceral fat. Unfortunately, these tests are expensive and require sophisticated equipment. Waist circumference, a more straightforward but more reliable method to measure abdominal adiposity, has become an increasingly important tool for classifying obesity (Hu, 2007). Numerous studies have shown that many obesity-related risk factors depend mainly on fat body distribution rather than excess weight *per se*. Hence, it is important to take into account how body fat is distributed in an individual, for example, between subcutaneous versus visceral (or intra-abdominal) fat. It is important to note that visceral fat, but not subcutaneous fat, is more associated with metabolic syndrome, which is further discussed below.

### Adipose Deposition

Adipose tissue is a complex, specialized, multicellular organ able to influence the function of almost all other organs. It is a loose connective tissue composed mostly of adipocytes, but which also contains the stromal-vascular fraction of cells (vascular endothelial cells, preadipocytes, and fibroblasts) as well as macrophages. Adipose tissue is very heterogeneous and, anatomically, consists of different fat depots with unique characteristics. This mentioned heterogeneity in adipose tissue is not only from an anatomical point of view but also from the characteristics of the tissue expansion, the regulation of lipid metabolism and also the pattern of secreted factors (collectively called “adipokines”) in a particular fat depot. All these characteristics bring with them metabolic consequences that impact the whole body, including the brain. It is known that each anatomical fat depot has a particular physiological role, which implies having specific metabolic and hormonal characteristics. As aforementioned, there is strong evidence showing that some fat depots are more robustly associated with disease development and outcomes.

In mammals, adipose tissue forms *in utero* just before birth and throughout life. Moreover, the continuous generation of new adipocytes has been shown in adult humans (Spalding et al., 2008). Unfortunately, little is known about adipocyte development. However, the study of adipose stem cell biology results extremely important for understanding adipose tissue development, expansion, migration, and maintenance. Adipose tissue is classified as white adipose tissue (WAT) and brown adipose tissue (BAT). These two main classes are molecularly and functionally different. WAT serves majorly as an energy store whereas BAT dissipates energy generating heat. WAT is spread throughout the body as subcutaneous and visceral fat. Subcutaneous WAT (sWAT) is a fat layer under the skin, with major depots in the upper and lower body. The upper body subcutaneous fat consists of superficial and deep abdominal fat, extremity fat, and, in the case of females, breast fat, whereas the lower body subcutaneous fat is mainly in the gluteofemoral region (Jensen, 2008; Kwok et al., 2016). Visceral WAT (vWAT) is around vital organs within the abdominal cavity and rib cage. Its major depots are the omental, mesenteric, epicardial, and mediastinal (Kwok et al., 2016). Numerous differences between sWAT and vWAT have been reported. For example, sWAT is heterogeneous and is composed mainly of unilocular adipocytes together with small multilocular adipocytes whereas vWAT looks more uniform and is composed mainly of large unilocular adipocytes (Tchernof et al., 2006; Tchkonia et al., 2007). It is believed that while increased sWAT deposition (known as a pear-shaped fat distribution) might protect against metabolic dysfunction, the increase in vWAT (known as an apple-shaped fat distribution) might increase the risk of metabolic disease (Grauer et al., 1984; Snijder et al., 2003a,b). Indeed, vWAT has been demonstrated to expand majorly by hypertrophy of preexisting adipocytes while sWAT expands by hyperplasia, i.e., the increase of the number of progenitor cells and subsequent differentiation. It is important to highlight that bigger hypertrophic adipocytes are usually associated with metabolic dysfunction. In line with this, very important in terms of metabolic disease is the fact that vWAT adipocytes are metabolically (i.e., lipolytically) more active than sWAT adipocytes, thus releasing more free fatty acids to the bloodstream (Wajchenberg, 2000; Hajer et al., 2008). vWAT is also associated with the release of pro-inflammatory adipokines (Fontana et al., 2007) and this would explain, at least partially, why central obesity is strongly linked with metabolic complications such as type-2 diabetes mellitus and cardiovascular disease, and many others caused by elevated free fatty acids (Jensen, 2008). Indeed, this characteristic of being metabolically less active than vWAT makes of sWAT a very important source of free fatty acids during food deprivation. It is important to highlight at this point that vWAT mass but not sWAT mass correlates with the development of insulin resistance (Chowdhury et al., 1994; Wajchenberg, 2000; Hoffstedt et al., 2018). A plethora of studies argue in favor of women better distributing fat in the periphery (compared to men having more central obesity) and thus having better metabolic health (Kwok et al., 2016).

Interesting experiments with transplantation of adipose tissue have been performed in animals, and they have been very useful for assessing the different functions and metabolic properties of the different fat depots. For example, transplantation of subcutaneous fat from donor mice into visceral fat site of recipient mice has shown to confer metabolic benefits in the latter, namely decrease in body weight and total fat, improvement of insulin sensitivity, and lowering of both insulinemia and glycemia (Tran et al., 2008). Unexpectedly, intraperitoneal transplantation of visceral fat from lean mice showed to improve insulin sensitivity, suggesting that the metabolic performance of a certain fat depot is more important as a metabolic risk factor than the anatomical location or the amount of fat itself (Konrad et al., 2007; Kwok et al., 2016). On the other hand, transplantation of visceral fat or subcutaneous fat into subcutaneous fat site has not shown to cause any alterations in body weight or metabolic profile, so it seems there are both donor and recipient site-specific factors that intervene in the final outcome (Tran et al., 2008). Other experiments have shown similar results in both autologous as well as heterotransplantation of subcutaneous fat into the intraabdominal cavity of diet-induced obese animals. In both cases, transplanted adipocytes showed to diminish their size, and insulin sensitivity, as well as serum lipid profile, showed to be improved, correcting almost all the metabolic parameters altered by obesity (Foster et al., 2013; Torres-Villalobos et al., 2016). Interestingly, transplantation of brown adipose tissue to the visceral cavity has also demonstrated to prevent weight gain and improve carbohydrate metabolism in high-fat diet-induced obese animals (Townsend and Tseng, 2012). All this evidence strongly suggests that fat cells from different depots do have different characteristics and thus can play protective or detrimental roles in metabolism.

### Obesity and Peripheral Health

Obesity is known as a chronic low-grade inflammatory disorder that results a triggering factor for many other metabolic and inflammatory disturbances. The obesity-linked inflammatory response includes many components of the classical inflammatory response, namely augmented secretion of circulating inflammatory factors, recruitment of leukocytes to inflamed tissues and organs, and activation of these leukocytes. However, the metainflammation observed in obesity has distinctive characteristics. For example, it is known that the chronic low-grade inflammation ends affecting the metabolic homeostasis in the long-term. Also, the multi-organ affection observed as the result of obesity-associated inflammation results to be unique (Lumeng and Saltiel, 2011). Adipose tissue, which was primarily thought to be a mere storage depot for triglycerides, is now considered an immune organ playing a vital role as a primary *in vivo* site of inflammation in obesity. Indeed, adipose tissue also plays a critical endocrine role due to the ever-increasing number of adipocyte-derived secretory factors that have been described.

#### Obesity, Adipokines, and Peripheral Inflammation

Substantial evidence supports that many of the circulating adipokines might be responsible for the peripheral inflammation observed in obese patients, including tumor necrosis factor α (TNFα), leptin, and various interleukins, among others. Importantly, the dysregulation of the adipokine secretion pattern has been linked to obesity and all the obesity-related metabolic disturbances such as cardiovascular disease, hypertension, type-2 diabetes, etc. Moreover, changes in either the amount or the quantity of the secreted adipokines are able to affect the various organ systems of the body vital for energy homeostasis. It is important to note that the weight loss-associated normalization of the adipokine secretion pattern is linked to the consequent normalization of different metabolic parameters, reinforcing the idea that adipokines are critical for the whole body metabolic homeostasis.

It is important to highlight that although secreted by adipose tissue, adipokines (except for leptin) are not solely secreted by adipocytes. Leukocytes, almost half of the non-adipocytes cell fraction in adipose tissue, are the source of classic adipokines such as IL-1, TNFα, visfatin, and resistin. Many other adipokines are produced and secreted by both adipocytes and leukocytes, such as adiponectin and IL-6. All fat depots are able to release adipose hormones, but visceral fat is considered to be the primary source of them. It has also been demonstrated that each fat depot has a particular pattern of adipokine expression (Dodson et al., 2014; Zhang et al., 2014). Interestingly, it has been shown that adipokines are also released by some particular places of the central nervous system where adipokine receptors are also expressed. Nonetheless, alterations of adipokine release during obesity and aging are attributed almost exclusively to changes in the structure and function of the adipose tissue (Kiliaan et al., 2014). A detailed description of each adipokine is beyond the scope of this review, but a few generalizations of the most important ones deserve mention.

Leptin is probably the most studied adipokine. It positively correlates with BMI (Friedman and Halaas, 1998; Lissner et al., 1999); however, obesity is considered a state of reduced leptin function. It is produced peripherally by WAT but exerts the bulk of its metabolic functions centrally, after crossing the blood brain barrier (BBB). However, it has been shown to be produced both in rodent and human brains (Morash et al., 1999; Wiesner et al., 1999; Brown et al., 2007; Wilkinson et al., 2007). It is well known that leptin action in the hypothalamus maintains body weight homeostasis in response to changes in the nutritional status. Leptin is considered the principal regulator of the “brain-gut-axis,” which provides a satiety signal through its action on hypothalamic leptin receptors (Konturek et al., 2004). Activation of these receptors suppresses food intake and promotes energy expenditure pathways (Tilg and Moschen, 2006; Simerly, 2008). It is worth highlighting that several hypothalamic neuropeptides have been shown to be produced by leptin-sensitive neurons and to act as neurotransmitters mediating leptin action (Xu and Tong, 2011). However, the specific neurotransmitter responsible for midbrain leptin action on feeding remains elusive. It has also been shown that leptin is able to regulate numerous inflammatory and immune processes, including cytokine expression and cell proliferation and death (O’Rourke, 2010). Very importantly, experiments of leptin receptor restoration in the brain of mice which completely lack the receptor function have shown the normalization of the metabolic parameters (de Luca et al., 2005). Interestingly, leptin signaling has also been suggested to be one of the circulating factors connecting obesity and the consequent reproductive dysfunction, being the reproduction defects reverted by pharmacological administration of leptin (Tong and Xu, 2012).

Adiponectin is an adipokine with insulin-sensitizing and anti-inflammatory effects produced exclusively by adipose tissue and suggested to be a visceral adiposity marker, due to the fact that several studies in humans have shown that visceral adipocytes secret more adiponectin than subcutaneous adipocytes (Lenchik et al., 2003; Ryan et al., 2003; Matsuzawa, 2007; Drolet et al., 2009). It exists as trimers, hexamers, and high-molecular-weight (HMW) multimeric complexes (Rutkowski and Scherer, 2014). Recent data indicate that the HMW complexes have predominant action in metabolic tissues (Achari and Jain, 2017). Unlike the majority of adipokines, adiponectin plasma levels correlate inversely with obesity, insulin resistance, and type-2 diabetes mellitus (Hotta et al., 2001; Kondo et al., 2002; Deng and Scherer, 2010). However, the decreased level of adiponectin in obesity is not clear yet. Physiological functions of adiponectin in the brain have been related majorly to food intake, energy expenditure, lipid and glucose metabolism, and body weight control (Kubota et al., 2007; Wen et al., 2010; Park et al., 2011). Interestingly, adiponectin physiological levels are generally higher in females than in males and decrease in both sexes as age increases (Ng and Chan, 2017). It should be mentioned that several studies have shown that the pharmacological reconstitution of adiponectin levels through drugs targeting adiponectin synthesis would help in the treatment of obesity and the associated diabetes and cardiovascular disease (Achari and Jain, 2017).

Resistin was first discovered to be secreted by adipocytes in rodents. However, in humans, it is predominantly expressed and secreted by macrophages. It is known that increased resistin levels are linked to the development of insulin resistance, diabetes mellitus, and cardiovascular disease. Moreover, resistin would promote endothelial dysfunction, vascular smooth muscle cell proliferation, arterial inflammation, and the generation of foam cells, thus contributing to the pathogenesis of atherosclerosis (Park et al., 2017). Circulating levels of resistin correlate directly with inflammatory markers such as C-reactive protein, TNFα, and IL-6 in patients with different metabolic disturbances (Park and Ahima, 2013).

Visfatin is also known as pre-B cell colony-enhancing factor (PBEF) or nicotinamide phosphoribosyltransferase (NAMPT), the latter due to the fact that it is the limiting enzyme in nicotinamide adenine dinucleotide (NAD) biosynthesis. This adipokine is expressed by different types of cells, including adipocytes, hepatocytes, and myocytes. However, in adipose tissue, it has been shown to be secreted majorly by infiltrating macrophages (Deng and Scherer, 2010). Visfatin is produced by visceral adipose tissue, and thus its production is increased in abdominal obesity. Interestingly, visfatin has been demonstrated to bind to insulin receptor and mimic insulin hypoglycemic effects, i.e., decreasing glucose release from the liver and increasing glucose uptake and utilization by peripheral tissues (Singla et al., 2010).

Apelin has been relatively recently classified as an adipokine since although it is produced and secreted by adipocytes, it is also expressed (together with its receptor) in the central nervous system and the cardiovascular system. Apelin has been related to the regulation of blood pressure, food intake, cell proliferation, and angiogenesis (Castan-Laurell et al., 2011). *In vitro* as well as *in vivo* studies have shown a strong relationship between apelin and insulin (Boucher et al., 2005). Moreover, apelin has been suggested to be the last protection before the appearance of obesity-associated metabolic disorders such as insulin resistance, type-2 diabetes, or cardiovascular disease (Castan-Laurell et al., 2005).

IL-6 is a pro-inflammatory cytokine synthesized and secreted by several cell types, including adipocytes and immune cells. Not only does IL-6 participate in inflammatory responses but it also controls feeding behavior at a hypothalamic level (Stenlöf et al., 2003). IL-6 circulating levels have been systematically reported to be augmented in obesity, being visceral fat secretion an important source of IL-6 thus linking the enlarged visceral fat with the existence of systemic inflammation in obese patients (Fontana et al., 2007). Importantly, IL-6 levels have been reported to normalize in morbidly obese patients who underwent bariatric surgery (Illán-Gómez et al., 2012).

TNFα is an inflammatory adipokine whose levels are increased in adipose tissue and plasma of both obese rodents and humans (O’Rourke, 2010). It was the first inflammatory adipokine associated with the onset and progression of insulin resistance (Hotamisligil and Spiegelman, 1994). It was first thought to be secreted by adipocytes, but today it is accepted that the bulk of TNFα is secreted by adipose tissue-resident macrophages. It has been shown that high levels of TNFα and IL-6 suppress the transcription of adiponectin thus connecting the role of visceral fat accumulation in adiponectin decreased secretion in obesity (Suganami et al., 2005).

In summary, adipose tissue, as an immune and endocrine organ, produces a wide variety of soluble factors collectively called adipokines. These molecules were initially associated uniquely to metabolic activities, but today it is known that they regulate numerous physiological and physiopathological events. Adipokines have pro- and anti-inflammatory properties and are considered fundamental circulating factors mediating the cross-talk between different organs and metabolic systems, thus integrating the systemic metabolism with immunity.

Before leaving the topic of adipokines, it is important to note that while adipokines have been primarily studied in terms of their links to adipose tissue, it is well established that non-adipose tissues produce and release adipokines. For example, muscle is known to produce and release several cytokines which are also produced and released by adipose tissue (Trayhurn et al., 2011; Görgens et al., 2015; Leal et al., 2018). Similarly, the liver is known to be an organ that is very much involved in the contribution of overall circulating adipokine levels. For example, leptin, adiponectin, and resistin have been shown to be locally produced by the liver (Marra et al., 2005). It is therefore important to understand that even circulating adipokine levels, in obese and non-obese individuals, is the result of the shared contributions of adipokine production from multiple tissues.

#### Obesity and Peripheral Metabolic Changes

As mentioned, the obesity-associated increase in the adipose tissue mass is linked to a change in the adipokine secretion pattern, thus causing what is known as “metainflammation,” which affects systemic metabolism. A common consequence of obesity is metabolic syndrome, a condition which is associated with pro-inflammatory states and which is considered to be a compilation of risk factors that predispose individuals to the development of cardiovascular disease and type-2 diabetes. The diagnosis of metabolic syndrome is made when any 3 of the 5 following risk factors are present: central obesity (enlarged waist circumference, defined according population-specific and country-specific criteria), high blood pressure (defined as systolic blood pressure ≥ 130 mm Hg or diastolic blood pressure ≥ 85 mm Hg), loss of glycemic control (elevated fasting glucose, defined as blood glucose > 100 mg/dl), low serum high-density lipoprotein (HDL) (defined as < 40 mg/dl in men and <50 mg/dl in women), and high serum triglycerides (defined as ≥ 150 mg/dl) (Lam and LeRoith, 2000). The existence of metabolic syndrome is well known to predispose an individual to diabetes and cardiovascular disease. It is important to know that metabolic syndrome also predisposes individuals to a number of other severe conditions including non-alcoholic fatty liver disease, non-alcoholic steatohepatitis, obstructive sleep apnea, and cancer, among others. Indeed, hyperleptinemia, hypoadiponectinemia, and insulin resistance are also widely linked to features of the metabolic syndrome.

Insulin resistance and type-2 diabetes mellitus are typical metabolic changes observed in obese patients. Insulin resistance implies impaired insulin-induced glucose uptake and metabolism in adipocytes and skeletal muscle, and impaired suppression of hepatic glucose production (Reaven, 1995). Insulin resistance is a key etiologic factor of type-2 diabetes but is also associated with a plethora of other pathophysiologic disorders including hypertension, hyperlipidemia, and atherosclerosis. Although several hypotheses about factors influencing insulin resistance coexist (including inflammation, mitochondrial dysfunction, hyperinsulinemia, lipotoxicity, oxidative stress, and endoplasmic reticulum stress), there is no consensus about a unifying mechanism for insulin resistance etiology (Ye, 2013). It is generally accepted that insulin resistance occurs first, with hyperinsulinemia as a pancreatic compensatory response, and after pancreatic failure to meet metabolic demands, hyperglycemia with hypoinsulinemia occurs. Importantly, central obesity precedes the development of insulin resistance, thus reinforcing the idea of visceral fat and its pro-inflammatory adipokines playing a key role in the pathophysiology of insulin resistance.

It is worth mentioning that although obesity is commonly associated with different metabolic abnormalities, 2–50% of obese adults are “metabolically healthy or metabolically normal” (the wide range is due to different criteria in the classification and also to the population studied) (Tiemann Luecking et al., 2015). The concept of “metabolically healthy obese” refers to obese people with normal metabolic risk profile (Karelis et al., 2005; Stefan et al., 2008). However, studies have previously shown that “metabolically healthy obese” individuals do have increased cardiovascular risk (Ärnloöv et al., 2010).

##### Effect of weight loss on metabolic endpoints of obesity

Reducing the amount of total body fat has been thought of as a strategy to diminish the impact of obesity and comorbidities on health. Weight loss interventions such as low-fat diets have been demonstrated to reduce many of the risk factors associated with obesity (Lee et al., 2011), as well as decrease all-cause mortality in obese adults. It is important to note that each reference to weight loss in this review is “intentional weight loss” and not involuntary weight loss. It is important to note that intentional weight reducing diets are routinely shown to be more effective in reducing blood pressure and improving dyslipidemia when combined with exercise (Schwingshackl et al., 2014). Interestingly, low-carbohydrate diets have been found to be more effective for reducing body weight than low-fat diets (Tobias et al., 2015). However, evidence supported by randomized controlled trials shows that most adults are unable to maintain weight loss (Wing and Phelan, 2005; Wadden et al., 2011; Ross et al., 2012). From this perspective, alternative approaches to diet-induced weight loss are needed, including the establishment of healthy eating and physical activity habits that may be more sustained over time. Bariatric surgery, also known as metabolic surgery, is associated with sustained weight loss, decreased cardiovascular risk factors and events, diminution in diabetes-linked microvascular complications, and improvement of obesity-associated comorbidities and overall survival (Christou et al., 2004; Sjöström et al., 2007; Sowemimo et al., 2007; Buchwald et al., 2009; Cummings and Rubino, 2018). Moreover, bariatric surgery has been shown to be superior to other weight loss-associated interventions in normalizing almost all the metabolic endpoints. However, studies with longer follow-up time (>5 years) are still needed, including studies that identify any potential long-term adverse effects following bariatric surgery (Busetto, 2014; Cummings and Rubino, 2018). Liposuction, which is mainly thought to remove subcutaneous fat without affecting visceral abdominal fat depot, is another common weight loss procedure. However, there is controversy about the impact of liposuction on obesity endpoints like insulin sensitivity. This controversy may be due in part to the fact that lipectomy of sWAT has been linked to the enlargement of vWAT (Benatti et al., 2012).

###### The complexity of weight loss in the elderly

As mentioned, there is a strong obesity-mortality association during adulthood. However, this association diminishes with age, and weight loss in older adults is not as beneficial as one might expect. Surprisingly, unintentional weight loss (more than 5% of body weight reduction in a year) in older adults is associated with increased morbidity and mortality (Gaddey and Holder, 2014). Although cachexia, i.e., the loss of muscle mass with or without fat loss, is thought to be the main responsible for these negative effects, the pathophysiology of unintentional weight loss remains unclear. It is well known that body composition changes with age, with fat mass gains until 65–70 years old, a characteristic peak in body weight around 60 years old, and gradual small decreases thereafter (Wallace and Schwartz, 2002). The ideal BMI in the elderly is considered (from a mortality point of view) to be of 25–30. At this point, it is worth to have in mind that although BMI in younger adults correlates quite well with total body fat, it does not in older adults and this might be an appropriate explication for the so-called “obesity paradox,” according to which obese old individuals have lower mortality than lean. The main reason is that not only there is a continuous loss of body muscle with age (without loss in body fat), but also the height is reduced due to spine-shortening as a consequence of age-related bone disease. In this sense, as mentioned before, waist circumference is a better index of adiposity, mainly because it correlates with abdominal body fat, which is the main contributor to metabolic disorders. Moreover, inactivity in older adults is usually accompanied by a loss in body muscle mass, a condition which may go unnoticed but which brings several functional consequences in the long-term period. This condition is known as sarcopenic obesity, and together with degenerative joint disease, it leads to the incapability to perform activities of daily living. Thus the impairment in daily function finally causes the development of frailty phenotype (which is generally present in the elderly but is greatly increased with obesity) with disability as a final outcome (Han et al., 2011).

Many observational studies have linked weight loss with increased risk of mortality (Yaari and Goldbourt, 1998; Knudtson et al., 2005; Sørensen et al., 2005; Shea et al., 2011). There are several causes of unintentional weight loss in the elderly (Gaddey and Holder, 2014). Only intentional weight loss in older people seems to lead to some clinical benefits, mainly due to the fact that unintentional weight loss is often associated with underlying subclinical illnesses. However, since intentional weight loss is linked to muscle mass loss and decreased bone mass (Waters et al., 2013), it appears that diet-induced weight loss should be accompanied by a program of physical activity which can potentially inhibit the muscle and bone loss associated with diet-induced weight loss (Shea et al., 2011). Lifestyle interventions that include diet plus exercise components have been shown to lead to a 10% weight loss with changes in physical function and metabolism (Waters et al., 2013). However, the clinical significance of these observations as well as long-term consequences of weight loss remains unclear.

#### Obesity and Peripheral Lipid Changes

Dyslipidemia is very common in obesity, reaching almost 70% of obese patients. The lipid abnormalities usually observed in obese patients are high levels of serum triglycerides, free fatty acids, very low-density lipoproteins (VLDL), Apo B, and non-HDL cholesterol (Franssen et al., 2011; Bays et al., 2013). HDL-cholesterol levels are typically found to be low together with HDL dysfunction. Regarding LDL-cholesterol, although in the normal range, the size of LDL particles is unbalanced, with more small pro-atherogenic LDL particles rather than large ones. Lipid changes in obesity have been shown to be strongly dependent on body fat distribution. For example, visceral adipose tissue and upper body subcutaneous adipose tissue have been related to high triglyceride and HDL cholesterol levels and insulin resistance, whereas lower body subcutaneous adipose tissue has been related to a healthier lipid profile (Feingold and Grunfeld, 2018).

All these obesity-related lipid abnormalities are frequent observations in metabolic syndrome and typically associated with the pro-inflammatory state described before. An important link between obesity, metabolic syndrome, and dyslipidemia appears to be insulin resistance in peripheral tissues. It has been shown that the increase in circulating free fatty acids associated with obesity contributes to several metabolic disturbances being insulin resistance probably the most important (Karpe et al., 2011). It is important to highlight, however, that the increase in free fatty acids is not only a consequence of insulin resistance but also contributes to its development (Lam and LeRoith, 2000). There are several reasons for increased free fatty acids in obesity: (1) enlarged adipose tissue resistant to the antilipolytic effect of insulin; (2) increased liver fatty acid *de novo* synthesis (Jacome-Sosa and Parks, 2014; Björnson et al., 2016; Xiao et al., 2016); (3) increased uptake of triglyceride-rich lipoproteins by the liver (Yu and Ginsberg, 2005; Dash et al., 2015; Björnson et al., 2016; Xiao et al., 2016). This increased free fatty acid flux finally exceeds adipose tissue lipid storage capacity, and free fatty acids begin to accumulate in the liver, pancreas, skeletal muscle, and heart, a condition known as “ectopic lipid deposition” which has several pathologic consequences. Lipids result cytotoxic to cells other than adipocytes. For example, lipid deposition in the pancreas has been involved in the development of diabetes whereas lipid deposition in the skeletal muscle has been associated with insulin resistance. Moreover, lipid accumulation in the liver defines steatosis. The hypothesis of adipose tissue protecting against ectopic accumulation of lipids (working as a metabolic sink) has been supported by observations in animals and humans with lipodystrophy, where the absence of adipose tissue is associated with generalized lipid ectopic deposition and insulin resistance (O’Rourke, 2010).

##### Effect of weight loss on peripheral lipids

The effect of weight loss on peripheral lipid profile has been extensively assessed. Serum fasting and non-fasting triglyceride levels have been demonstrated to be reduced by weight loss (Chan et al., 2002; James et al., 2003). On average, a 3-kg weight loss represents a reduction of 15 mg/dl in triglyceride levels. Indeed, LDL levels are also usually decreased by weight loss, and HDL levels are usually increased: a 5- to 8-kg weight loss is associated with a decrease of 5 mg/dl in LDL and an increase of 2–3 mg/dl of HDL (Ebbert et al., 2014). However, considerable variability in the results has been observed, and the improvement of dyslipidemia is not always the final outcome. On the other hand, the type of weight-loss diet followed does impact on lipid profiles. It is still unclear which factors associated with weight loss are predictive of the change in lipid profile. Several studies have shown a poor correlation between lipid profile improvement and the degree of weight loss, and a threshold effect has been suggested when the lipid profile resulted improved with a minimal weight loss but when no further effects were observed with a larger weight loss (Kelly and Jones, 1986; Busetto et al., 2000).

Interestingly, metabolic surgery impacts positively on serum lipid levels as a consequence of weight loss. Normalization of serum lipid profile is a common observation after gastric bypass surgery. In a meta-analysis of 11 randomized clinical trials comparing surgical versus non-surgical treatment of morbid obesity, bariatric surgery was found to be associated with more significant weight loss, remission of metabolic syndrome, and improvement in lipid profiles with the consequent decrease in medication requirements (Gloy et al., 2013; Koliaki et al., 2017). A 5-year follow-up study compared patients who received medical therapy alone with patients who underwent surgical therapy. Surgical patients were found to achieve the greatest health benefits, with a more significant reduction in triglyceride levels and a more considerable increase in HDL with respect to patients who received medical treatment alone (Schauer et al., 2017). It is worth mentioning that the beneficial effects of bariatric surgery have been analyzed in the short-term period and up to 5 years after the intervention; however, the long-term effects remain elusive.

## Effects of Obesity-Associated Fat Deposition and Circulating Factors on the Various Non-CNS Organs of the Body

**FIGURE 2:**
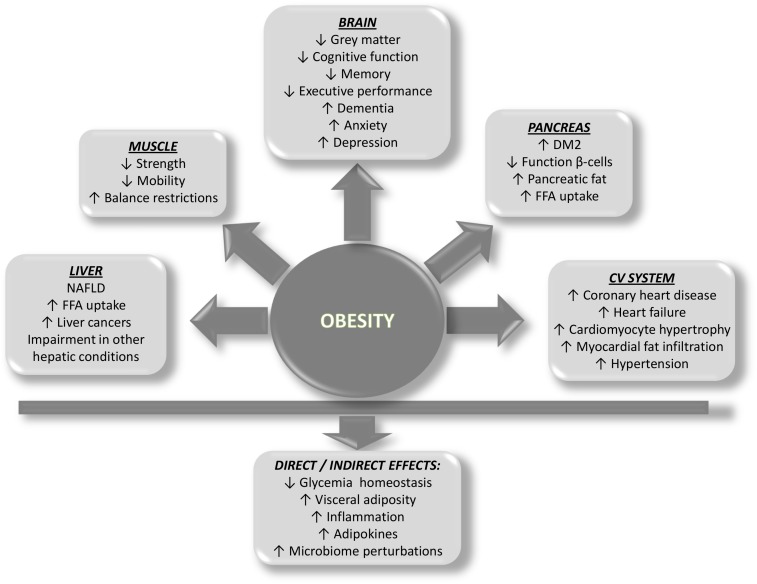
Scheme showing the predicted impact of obesity on organ systems and the direct and indirect effects on health.

Although the cause of obesity is mainly attributed to energy imbalance, the etiology of obesity is multifactorial, including genetic, psychological, economic, environmental, social, and physiological factors, only to cite some (Wright and Aronne, 2012). Whatever etiopathogenesis of obesity is considered, several organs are damaged as a consequence of the development of obesity, including the pancreas, liver, muscle, and the cardiovascular system. A brief outline of how ectopic fat deposition and the concomitant obesity-related circulating factors contribute to disease of each organ is provided (Figure 2).

### Pancreas

The ectopic fat accumulation in the pancreatic gland is usually referred to as pancreatic steatosis. Intra- and interlobular adipocytes, acinar cell fat, as well as islet fat content have been found to be augmented in obesity (Pinnick et al., 2008; Lee et al., 2010). A close relationship between insulin resistance and pancreatic steatosis has been described. As it is known, pancreatic β-cells usually produce and release insulin to control glucose homeostasis. Under the condition of insulin resistance, pancreatic β-cells increase the production and release of insulin to maintain the normal glycemia. However, patients predisposed to type-2 diabetes fail to secrete enough insulin to meet the metabolic demand (due to insulin resistance in several tissues) and type-2 diabetes occurs (Poitout, 2004). Different stages in the development of type-2 diabetes have been well characterized. In the first stage, there is insulin hypersecretion, which allows for normal glycemic control. In contrast, during the second stage of type-2 diabetes, there is a failure of β-cells to secrete sufficient insulin for glycemic control, and thus hyperglycemia is manifest. However, it is a matter of debate whether dysfunction precedes or follows the loss of β-cells in obesity-linked diabetes mellitus (Alarcon et al., 2016). Whatever the origin of obesity-associated diabetes, there is a failure in insulin production and/or the secretory capacity of β-cells. Interestingly, *in vitro* studies have shown that β-cells have an exceptional capacity to synthesize significant amounts of insulin even in obesity-like conditions, and that this insulin is readily available for secretion. It is believed that fat accumulation in the pancreatic islets would be responsible for, at least in part, the decreased insulin secretion of the second stage of type-2 diabetes, since ectopic fat deposits in the pancreas have been reported to cause β-cell dysfunction, both directly through lipotoxicity exerted by free fatty acids and indirectly through activation of inflammatory pathways (Le et al., 2011; Pezzilli and Calculli, 2014). Moreover, evidence suggests that lipid toxicity to pancreatic β-cells is a long-term process and it takes around a decade before diabetes is diagnosed (Oakes et al., 1997). On the other hand, studies performed in morbidly obese humans have shown that obesity and peripheral insulin resistance are also associated with substantial changes in pancreatic metabolism and pancreatic blood flow, together with β-cell dysfunction. Interestingly, as a consequence of insulin resistance in adipose tissue, increased free fatty acids have been shown to be delivered from the bloodstream in morbidly obese individuals, and these people clearly show a shift in the preferred pancreatic energy substrate, with lipid usage being predominant over glucose usage (Honka et al., 2014). The possibility of these observations (pancreatic metabolic shift and defect in blood flow) to be reverted by weight loss is still unclear.

In addition, the changes in circulating adipokines constitute an important link between the excessive adiposity in obesity, insulin resistance, and β-cell failure, mainly due to the fact that several adipokines have demonstrated to affect both the function and survival of β-cells thus deteriorating the function of the pancreas and contributing to acute and chronic pancreatitis as well as pancreatic cancer (Zhao et al., 2006; Gumbs, 2008). Leptin, adiponectin, resistin, and visfatin are the most important adipokines that would participate in the pathogenesis of pancreatic diseases (Biernacka and Małecka-Panas, 2015). Beyond the previously mentioned physiological roles of leptin, this adipokine has also been shown to reduce insulin secretion. It has also been demonstrated to regulate the inflammatory response, thus protecting the pancreas from some kinds of damage by reducing TNFα and increasing IL-4 production (Jaworek et al., 2002). Indeed, adiponectin has been shown to play anti-diabetic and anti-inflammatory roles. Regarding the role of adipokines and pancreatic disease, it is thought that the higher risk of acute pancreatitis in obese people would come from the increased adipokine-producer visceral fat in the surroundings of the pancreas (Biernacka and Małecka-Panas, 2015). However, the precise role of adipokines in acute and chronic pancreatitis, as well as in pancreatic cancer, is unclear yet and needs to be further investigated. Controversial results regarding the functions of leptin, visfatin, adiponectin, and resistin have been found.

In summary, obesity-linked type-2 diabetes is characterized by the decreased number and function of pancreatic β-cells. The impairment in β-cell function, as well as the number of β-cells, has been related to lipotoxicity (with the concomitant increased oxidative and endoplasmic reticulum stress) and the adipokine-induced inflammation processes (Halban et al., 2014).

### Liver

Obesity has been involved as a risk factor at different stages of liver disease (Manne and Saab, 2014), not only causing non-alcoholic fatty liver disease (NAFLD) but also impairing the general state of patients with other preexisting conditions such as viral hepatitis. NAFLD is an important metabolic risk factor defined as a higher than 5% intracytoplasmic fat deposition in the hepatocyte in the absence of alcohol consumption, toxin exposure or viral disease (Tiniakos et al., 2010; Calvo et al., 2015). However, fat deposition is not the only observation of NAFLD, an inflammatory process coexists, with hepatocellular ballooning injury that can lead to fibrosis and cirrhosis (Brunt et al., 1999). Since triglycerides have been shown to accumulate in the liver through different manners, there is not a unique mechanism by which obesity could lead to NAFLD. Moreover, beyond the role of triglycerides, insulin resistance does have an important role in the development of fatty liver (Marchesini et al., 1999; Sanyal et al., 2001; Willner et al., 2001; Pagano et al., 2002). Free fatty acid uptake by the liver not only leads to hepatic steatosis but also produces hepatic toxicity by oxidative stress-dependent mechanisms (Manne and Saab, 2014). In this connection, it is thought that hepatic accumulation of triglycerides occurs first (hepatic steatosis or fatty liver) and then, since fatty liver is more prone to suffer oxidative injury, it evolves to steatohepatitis (Qureshi and Abrams, 2007).

The link between obesity and NAFLD has been explained by different hypotheses which take into consideration the portal hypothesis, the endocrine role of adipokines, and many observations from lipodystrophic states. It has been mentioned that visceral adipocytes constitute an important source of fatty acids and other factors entering the portal circulation (Qureshi and Abrams, 2007). The portal hypothesis supports the idea that the increased hepatic uptake of fatty acids coming from an enlarged visceral adipose tissue leads to decreased hepatic insulin clearance and thus increased circulating insulin. Indeed, fatty acids stimulate hepatic gluconeogenesis, triglyceride synthesis, and hepatic glucose output (by altering insulin signaling) (Kahn and Flier, 2000). On the other hand, adipokines are also involved in the development of NAFLD during obesity. For example, in addition to all the general effects of leptin, this adipokine has been shown to better liver enzymes and hepatic fat content, thus attenuating different manifestations of fatty liver in patients with lipoatrophy and metabolic syndrome (Lee et al., 2006). In obese NAFLD patients, leptin levels correlate with the severity of fatty liver, thus suggesting the presence of leptin resistance, probably due to a failure in leptin signaling. Low levels of adiponectin have been found in NAFLD patients, probably due to the concomitant high levels of IL-6 and TNFα found, both of which inhibit adiponectin expression. Therapy with adiponectin administration has shown to improve insulin resistance in animal models of obesity; however, in lipodystrophic animal cases, the complete reversal of insulin resistance requires the co-administration of leptin. Adipose tissue-derived TNFα and IL-6 cause the activation of Kupffer cells which leads to hepatic fibrogenesis. Moreover, TNFα has been shown to be also produced by the Kupffer cells, playing a key role in the pathogenesis of NAFLD (Qureshi and Abrams, 2007). Additionally, fatty liver, accompanied by insulin resistance and diabetes, is usually observed in lipodystrophic patients, where the fatty liver usually progresses to cirrhosis. Among different explanations proposed, reduced adiponectin and leptin levels are thought to be responsible for the presence of NAFLD in lipodystrophic individuals.

Not only has obesity been related to the development and progression of NAFLD, but also with the impairment of other hepatic conditions, being obesity considered as a strong risk factor for different liver cancers. Very interestingly, the mere losing weight of obese patients has been shown to be sufficient not only to improve the results of several hepatic treatments (Nobili et al., 2011) but also to increase insulin sensitivity and decrease hepatic triglyceride and free fatty acid uptake by liver (Viljanen et al., 2009; Manne and Saab, 2014).

### Muscle

As a general picture, obesity is linked to functional limitations in muscle performance and increased probability of developing a functional disability related to strength, mobility, postural, and dynamic balance restrictions. It is known that obese people, regardless of age, have greater absolute maximum muscle strength in anti-gravity muscles compared to non-obese counterparts (this is valid only for lower limbs, since upper limb strength reveals no statistical difference between obese and normal-weight people) (Maffiuletti et al., 2007, 2008; Delmonico et al., 2009; Abdelmoula et al., 2012; Tomlinson et al., 2016). This observation has been interpreted as adiposity being a chronic overload stimulus for muscles, thus making muscles stronger and bigger. However, it is noteworthy that when muscular strength is normalized to total body mass, the obese people seem to be overall weaker than their lean control individuals. Notwithstanding, the existing literature shows considerable controversy on this matter (Tomlinson et al., 2016). For this reason, the real effect of obesity on skeletal muscle size, structure, and function remains elusive. Although there is no consensus regarding accurate measures of obesity-associated muscle damage or quality, it is worth mentioning that obesity does have been shown to generate a negative impact on skeletal musculature through adolescence to young and old adulthood (Blimkie et al., 1990; Rolland et al., 2004; Zoico et al., 2004; Maffiuletti et al., 2007, 2008). Obviously, the relevance for reduced muscle performance is higher for older people, as they are generally affected by reduced functional capacity. This includes impairments in walking, impaired ability to go up and downstairs, and difficulty with rising from chair or bed. Similarly, there is an augmented risk to suffer joint pathologies, such as knee osteoarthritis, due to joint overload and reduced muscle strength (Cooper et al., 1998; Rolland et al., 2009; LaRoche et al., 2011; Maden-Wilkinson et al., 2015). All these conditions represent a clear sign of poor quality of life for older people, which is worsened by obesity. Further work is needed to systematically investigate whether body fat percentage *per se* may be related to agonist muscle activation and antagonist co-activation and/or morphological and architectural muscle characteristics.

In connection to ectopic fat deposition, the accumulation of triglycerides intra-myocellularly and inter-myocellularly is known to cause lipotoxicity, insulin resistance, and impaired glucose metabolism. The increased flux of fatty acids to the myocyte appears to be the link between muscle fat infiltration and insulin resistance and altered glucose metabolism. It has been shown that when mitochondrial β-oxidation is overwhelmed due to an excess of free fatty acids entering the myocyte, metabolic intermediaries of fatty acids accumulate and finally impair insulin signaling (Moro et al., 2008; Ghosh, 2014). It has been shown that skeletal muscle fat infiltration, together with sarcopenia, is able to have not only metabolic consequences but also to impair the daily living, by diminishing mobility (Katsanos and Mandarino, 2011).

The effect of adipokines on the skeletal muscle has emerged as an important area of research. It has been shown that adipokines including leptin, adiponectin, visfatin, and resistin are able to affect muscle insulin sensitivity (Nicholson et al., 2018). There are several *in vitro* studies about the role of adipokines in the skeletal muscle metabolism and insulin sensitivity. However, those studies have been carried out mainly using rodent skeletal muscle cells that are known to have different fiber composition and different metabolic characteristics than human skeletal muscle cells. This may be the reason why reports about the role of adipokines in muscle function and insulin resistance result controversial.

Beyond the effect of inflammation-associated adipokines on skeletal muscle, it is worth mentioning that under obesity conditions, the muscle becomes an inflammatory organ itself, able to secrete several circulating factors (known as myokines) that can act in either autocrine, paracrine or endocrine manner, thus affecting the metabolism of both muscle and other organs. The muscle is able to secrete several hundred of factors in response to contraction, which became a whole new paradigm for understanding the communication between muscles and the various organ systems, including adipose tissue, liver, pancreas, and brain. It is worth to mention that some myokines exert their functions in the muscle itself and have been suggested to regulate skeletal muscle growth, repair, maintenance, and regeneration, in addition to mediating the health benefits of exercise (for review see Pedersen, 2013).

### Cardiovascular System

Obesity, at the population level, has been considered as a risk factor for the development of cardiovascular diseases such as coronary heart disease and heart failure. Obesity-associated transition from asymptomatic subclinical left ventricle changes to overt dilated cardiomyopathy, irrespective of the coexistence of hypertension or diabetes mellitus, has been shown (Wong and Marwick, 2007). Importantly, body fat distribution has been found to be more important than total fat composition on left ventricle adaptations to obesity, with excessive visceral fat causing adverse hemodynamics, concentric left ventricle remodeling, lower cardiac output, and higher systemic vascular resistance. Lower-body subcutaneous fat has been found to cause eccentric left ventricle remodeling, higher cardiac output, and lower systemic vascular resistance, thus suggesting a protective role for subcutaneous adipose tissue, highlighting the importance of adipose tissue quality and function more than just the amount of body fat *per se* (Kim et al., 2016). In this regard, one hypothesis holds that the presence of insulin-sensitive subcutaneous adipose tissue protects the individual from ectopic accumulation of lipids and the development of metabolic syndrome (Kim et al., 2016). Cardiomyocyte hypertrophy and myocardial fat infiltration have also been demonstrated in obesity (Ommen and Lopez-Jimenez, 2013; Samanta et al., 2015). In this sense, obesity-triggered ectopic fat deposition is considered as a predictive risk factor for cardiovascular disease. The above mentioned ectopic fat accumulation in the liver and muscle (together with the associated inflammation) in particular constitutes a cardiovascular risk due to the associated insulin resistance and altered lipid and glucose metabolism. Fat surrounding the heart and blood vessels and within the renal sinus has been linked to local toxic effects by several lines of evidence. The damaging effect of ectopic fat in the cardiovascular system has been attributed to two main mechanisms: (1) fat deposition around the heart (pericardial or epicardial fat) and coronary arteries, (2) lipid accumulation within the cardiomyocyte. Pericardial, perivascular, pericoronary, and myocardial fat accumulation may lead to injury in blood vessels and heart directly by lipotoxicity and indirectly by cytokine secretion (Lim and Meigs, 2014). Fat in the neck is the only fat depot in the upper-body that is considered as an additional cardiovascular risk, and it has been found to positively correlate with insulin resistance, visceral fat content, and metabolic syndrome (Ben-Noun and Laor, 2003; Preis et al., 2010). Interestingly, pericardial fat has been proposed to play roles in support and mechanical purpose (for example, attenuation of vascular tension and torsion). However, this fat depot, when gets considerably enlarged in obesity conditions, represents a mechanical hindrance for the beating heart, thus altering cardiac size and performance (Iacobellis, 2009). In this connection, according to the Framingham Heart Study, pericardial fat is associated with coronary artery calcification and impaired cardiac function and conduction (Rosito et al., 2008). Accumulated fat around the coronary arteries and the heart appears to promote the atherosclerosis process, being associated with oxidative stress-related mechanisms. Myocardial fat accumulation has been associated with increased left ventricle mass, myocardiopathy and heart failure, mainly due to lipid-caused apoptosis of cardiomyocytes and the consequent cardiac dysfunction (Szczepaniak et al., 2003; Lim and Meigs, 2014).

A vast body of experimental, epidemiological, and clinical evidence supports the idea that obesity results harmful for both cardiovascular structure and function, mainly due to increased inflammation caused by deregulated adipokine production by a dysfunctional adipose tissue. In line with this, active endocrine and paracrine activity of cardiac ectopic fat depots within the cardiovascular system may be greatly responsible for insulin resistance and the atherosclerosis process. Moreover, leptin, adiponectin, resistin, visfatin, omentin, IL-1, IL-6, plasminogen activator inhibitor-1, and TNFα, among several other circulating factors, have been reported to signal to the myocardium through either paracrine or autocrine pathways (Iacobellis, 2015). Also, some anti-inflammatory factors secreted from perivascular fat (adiponectin, adrenomedullin, and omentin) have been demonstrated to play a key protective role in the regulation of the arterial vascular tone (vasodilation), decreasing oxidative stress, improving endothelial function, and increasing insulin sensitivity (Sacks and Fain, 2007; Piché and Poirier, 2018). It is important to highlight that secretion of pro-inflammatory adipokines is not only due to adipocyte secretion but also to secretion coming from adipose tissue-infiltrated macrophages (Chatterjee et al., 2009).

In summary, beyond the contribution of visceral fat-secreted adipokines to cardiovascular disease, the presence of cardiac ectopic fat does also contribute: firstly, due to mechanical functions, and secondly, due to cardiac ectopic fat-released adipokines which would link the ectopic cardiac fat depot, the vasculature, and the myocardium, thus playing key roles in the pathogenesis of cardiovascular disease.

## The “Obese” Brain

This section of the review is dedicated exclusively to the brain in the context of obesity. We will approach this topic from a variety of perspectives, including the anatomical and functional changes observed in the brain of obese individuals, the effects of obesity-associated circulating factors on the brain, the effects of obesity-associated morbidities on the brain and, last but not least, the effects of obesity-associated inflammation on the brain.

### Anatomical Aspects

Differences in both gray and white matter have been reported in obese individuals compared to their normal-weight counterparts. Regarding the gray matter, it has been shown that it is reduced in brain regions such as the hippocampus, prefrontal cortex, and other subcortical regions in the context of obesity (Stillman et al., 2017). Interestingly, these differences have been attributed exclusively to excessive adiposity, since they have been shown to be still present even after controlling obesity-associated conditions, including diabetes (Raji et al., 2009). Hippocampal atrophy is of particular importance since it has been related to Alzheimer’s disease (AD) (Elias et al., 2000). Reduction in the volume of gray matter has been quite well established in several other brain regions of obese individuals using a variety of methodologies (Pannacciulli et al., 2006; Raji et al., 2009; Medic et al., 2016). There are studies reporting both obesity-associated reductions, as well as increases, in white matter in the context of obesity (Pannacciulli et al., 2006; Raji et al., 2009; Debette et al., 2010; Driscoll et al., 2012; van Bloemendaal et al., 2016).

### Cognitive Function

Obesity and metabolic syndrome have undoubtedly been linked to deterioration in cognitive function. Moreover, clinical data have shown that obesity and diabetes mellitus are linked not only to cognitive decline but also to other brain disorders such as dementia, anxiety, and depression (Simon et al., 2006; Riederer et al., 2017; Sanderlin et al., 2017). Due to the difficulty to dissect the impact of each component of the obesity-associated altered metabolism on neuronal performance, it is assumed that brain structural changes, as well as the consequent cognitive impairment, are the result of the synergistic interplay between the different obesity-induced risk factors (Yaffe, 2007; Yates et al., 2012). Several models have been proposed that include the involvement of oxidative stress, inflammation, and abnormal brain lipid metabolism (Yates et al., 2012). Peripheral insulin resistance has been shown to be accompanied with cognitive decline, mainly in memory and executive performance (Heni et al., 2015; Mainardi et al., 2015; Cheke et al., 2017).

Several studies have reported that obesity in midlife is associated with increased risk of mild cognitive impairment, altered executive functioning and short-term memory, and dementia (Kivipelto et al., 2005; Cournot et al., 2006; Whitmer et al., 2008; Sabia et al., 2009; Nguyen et al., 2014). Similar results have been shown in studies carried out in animal models of high-fat diet-induced obesity (Murray et al., 2009; McNeilly et al., 2011; Nguyen et al., 2014). In contrast, the association between obesity late in life and cognitive function is less clear. A recent and important study of more than 10000 men and women, followed for up to 28 years, has examined the link between obesity and cognitive change. In this study, participants were assessed for BMI, waist circumference, signs of dementia, as well as cognitive decline (Singh-Manoux et al., 2018). This study identified that obesity (BMI > 30) at age 50 years is a risk factor for dementia, whereas obesity was not a dementia risk factor at ages 60 and 70 years. This difference may be due to the fact that BMI starts to decline several years before the diagnosis of dementia (Singh-Manoux et al., 2018). These findings could explain, at least in part, the situation known as “obesity paradox” in which underweight older people consistently show an increased risk of dementia while people having normal BMI or even being overweight in the elderly do not.

### Effects of Obesity-Associated Circulating Factors on the Brain

Vast epidemiological evidence supports a link between diabetes mellitus and cognitive dysfunction (Gudala et al., 2013; Koekkoek et al., 2015; Zhao et al., 2018). However, it should be mentioned that this association, as well as the severity of cognitive decline, may vary according to the type of diabetes and the age diabetes starts. Loss in glycemic control, evidenced by increased circulating HbA1c levels, has been found to be a risk factor for cognitive dysfunction, with behavioral and psychological manifestations (Sakurai et al., 2014). However, the Leiden 85-plus Study, which prospectively evaluated 599 individuals of ∼85 years of age, reported that HbA1c concentrations were not associated with cognitive dysfunction (van den Berg et al., 2006). Clinical evidence has suggested that the duration of diabetes alone may not influence cognitive performance if glycemia is properly controlled over time (West et al., 2014). Interestingly, beyond the chronically high glucose levels, blood glucose peaks have been related to both cognitive impairment and increased risk of dementia (Geijselaers et al., 2015; Rawlings et al., 2017). Additionally, observational studies have shown beneficial effects of some glucose-lowering treatments on cognitive performance. For example, metformin has been shown to improve cognitive performance in US diabetic veterans (Orkaby et al., 2017).

Leptin deficiency has been linked to alterations in brain volume and structure, and these alterations have been shown to be reversed by external leptin administration (Matochik et al., 2005; London et al., 2011). Leptin has been shown to have a direct impact on the hypothalamic nuclei which are responsible for the production of both orexigenic and anorexigenic peptides (Qi et al., 2004; Kishi and Elmquist, 2005). Indeed, leptin has been demonstrated to exert neurotrophic actions during the development of the hypothalamus, stimulating the growth of axons from the arcuate nucleus to other regions that control energy homeostasis, thus participating in the development of feeding circuits. Interestingly, this developmental activity of leptin has been shown to depend on timing and duration of leptin exposure (Bouret et al., 2004, 2012; Bouyer and Simerly, 2013; Kamitakahara et al., 2018). Leptin has also been related to presynaptic neurotransmitter release and postsynaptic neurotransmitter sensitivity, and to the processes of memory and cognition, especially to hypothalamic and hippocampal functions (Fewlass et al., 2004; Davidson et al., 2005; Harvey et al., 2005; Oomura et al., 2010). Alterations in hippocampal structure and function have been reported in animals with congenital leptin deficit, supporting a role for leptin in hippocampal development and function (Li et al., 2002; Dhar et al., 2014). Neurodegeneration, neurogenesis, synaptic plasticity as well as memory consolidation have been shown to be influenced by leptin action on the hippocampus (Doherty, 2011). Also, leptin has been shown to enhance cognition through the regulation of hippocampal function. Both *in vivo* and *in vitro* studies (the latter in hippocampal slices) have shown that exogenous leptin is able to induce long-term potentiation (Shanley et al., 2001; Wayner et al., 2004; Luo et al., 2015). Other *in vitro* studies have shown that leptin is able to induce synapse formation in cultured hippocampal neurons, thus providing a possible explanation for the long-term potentiation observed after leptin administration. Studies in humans have shown that high leptin levels are negatively correlated with late-in-life dementia risk (Harvey et al., 2005; Lieb et al., 2009). Moreover, leptin has been shown to reduce extracellular levels of amyloid beta peptide (Aβ; whose deposition is pathognomonic of AD) both *in vivo* and *in vitro* (Fewlass et al., 2004). For these reasons, the elevation of leptin has been suggested as a therapeutic alternative for dementia (Fewlass et al., 2004; Harvey et al., 2005; Irving and Harvey, 2013; McGregor and Harvey, 2018). Although animal studies are promising, further research is needed to assess whether these findings apply to human beings.

Due to undetectable levels of adiponectin in the cerebrospinal fluid (CSF), it was first thought that this hormone was not able to cross the BBB (Pan et al., 2006; Spranger et al., 2006). However, several studies have shown that intravenous injection of adiponectin leads to detectable levels of the hormone in the CSF of patients with unspecified neurological disorders (Kos et al., 2007; Kusminski et al., 2007; Neumeier et al., 2007). Indeed, as no HMW adiponectin has been detected in the CSF, it is now believed that only smaller forms of the adiponectin hormone can cross the BBB (Kusminski et al., 2007; Schulz et al., 2011). Thus, the origin of brain associated adiponectin is still a matter of debate. Adiponectin plasma levels correlate inversely with obesity, insulin resistance and type-2 diabetes mellitus (Hotta et al., 2001; Kondo et al., 2002), with adiponectin levels in the CSF being 1000-fold lower than the plasma levels (Kos et al., 2007; Kusminski et al., 2007). Adiponectin has been shown to regulate proliferation, neurogenesis, and branching of hippocampal neural stem cells (Zhang et al., 2011, 2016; Yau et al., 2014). Also, it has been shown to exert a neuroprotective role against Aβ-induced oxidative stress *in vitro* (Ng and Chan, 2017). Adiponectin deficiency in mice has shown to cause memory and spatial learning impairment, anxiety, and impaired fear conditioning, symptoms that are probably associated to the decreased synaptic protein levels, increased neuronal apoptosis and impaired insulin signaling found in those animals (Ng et al., 2016). Also, adiponectin-deficient mice have shown to suffer larger brain infarctions after ischemia and reperfusion compared with control animals, and adiponectin administration has shown to reduce the infarction size. Thus neuroprotective effects have been attributed to this adipokine (Nishimura et al., 2008). Adiponectin physiological levels are generally higher in females than in males and decrease in both sexes as age increases (Ng and Chan, 2017). However, among women, those with higher plasma levels of adiponectin have shown to exhibit poorer performance in language and global cognition and to coincide with greater mild cognitive impairment diagnosis (Wennberg et al., 2016). Nevertheless, more studies are necessary to conclusively affirm that higher adiponectin plasma level is a trustable predictor of cognitive decline. Patients with AD have been observed to have decreased levels of adiponectin in CSF, compared to those found in healthy controls or even to patients with mild cognitive impairment. However, adiponectin levels have been found to be increased in plasma of patients with mild cognitive impairment and AD, compared to that in controls. Thus, a loss of function of adiponectin signaling has been suggested to occur in the pathogenesis of AD (Waragai et al., 2017).

As mentioned before, the increased circulating levels of pro-inflammatory cytokines participate in obesity-induced systemic inflammation. This systemic inflammation may participate in the development of cognitive decline and dementia. For example, IL-1β and IL-6 have been shown to disrupt cognition- and memory-related neuronal circuits (Gemma and Bickford, 2007). Increased plasma levels of C-reactive protein and IL-6 have been identified in a meta-analysis performed by Koyama et al. (2013). Peripheral cytokines have been shown to induce local production of cytokines in the brain (Dantzer et al., 2008).

### Effects of Obesity-Associated Morbidities on the Brain

It is important to highlight that all the obesity-associated morbidities mentioned before (cardiovascular disease, diabetes, atherosclerosis, etc.) do impact on brain health. Obesity-derived vascular problems, such as atherosclerosis and arteriosclerosis, which are systemic diseases, are known to affect the steady blood flow of vessels that feed the brain, thus contributing to cognitive impairment or even stroke, where large areas of the brain die due to the stop in the blood flow of a major brain artery caused by a blood clot. Vascular dementia has been shown to be caused by cerebrovascular disease, and compelling evidence has shown that cerebrovascular disease may be initiated by obesity (Gorelick et al., 2011; Zlokovic, 2011). However, many aspects of the association between obesity and cerebrovascular disease are still poorly defined. Also, epidemiological studies have shown that cardiovascular risk factors such as obesity, hypertension, diabetes, and low physical activity negatively affect brain performance (Wolf, 2012; Yano et al., 2014). A longitudinal study from Gustafson and coworkers has shown lower BBB integrity in overweight or obese individuals, compared to normal-weight controls (Gustafson et al., 2007). Similar evidence has been presented from rodent studies (Kanoski et al., 2010; Davidson et al., 2012). Indeed, irregular heartbeat conditions such as arrhythmia or atrial fibrillation, as well as obstructive sleep apnea (both highly prevalent in obese individuals), have been linked to increased risk of ischemic stroke and dementia development (Zhang et al., 2015). Interestingly, several studies have reported that obese people who survive to a first stroke event usually have improved subsequent cerebrovascular disease and mortality, as part of the previously mentioned “obesity paradox.” This could come in line with the fact that obese people tend to suffer more lacunar-type of stroke, which is generally of faster recovery and better prognosis (Letra and Seiça, 2017).

Epidemiological studies have linked type-2 diabetes mellitus with cognitive impairment and dementia, with insulin resistance and hyperglycemia as the probable mechanistic links (Ott et al., 1996; Peila et al., 2002). Similarly, several cross-sectional studies have confirmed the association between insulin resistance and cognitive decline (van den Berg et al., 2006; Young et al., 2006; Ekblad et al., 2017). Hyperglycemia has been associated with poor cognitive outcomes both in cross-sectional studies (Yaffe et al., 2012) as well as in prospective studies (Prickett et al., 2015). A very recent 6-year follow-up study from Hong and coworkers has found that insulin resistance is associated with diminished cognitive performance in older individuals (Kong et al., 2018). Also, data from prospective studies have shown that individuals with type-2 diabetes exhibit poorer performance in information-processing speed, memory, attention, and executive function compared to controls (Hassing et al., 2004; van den Berg et al., 2010; Moheet et al., 2015). Longitudinal and cross-sectional studies have undoubtedly demonstrated a relationship between diabetes and mild/moderate cognitive dysfunction in type-2 diabetes, but less is known about the strength of association between diabetes and dementia. Systematic review and meta-analysis performed by Biessels and coworkers have shown an increase of 50–100% in the risk of dementia in people with type-2 diabetes, compared with people without diabetes (Biessels et al., 2006). However, the evidence is controversial, and further interventional studies are needed to evaluate the effect of controlling insulin resistance and diabetes on cognitive dysfunction.

Interestingly, obesity comorbidities have been shown to participate in the onset and progression of neurodegenerative diseases such as AD. The complete mechanisms by which obesity influences the risk of AD is not entirely clear yet. However, epidemiological studies have demonstrated that type-2 diabetes increases the risk of AD (Profenno et al., 2010). It is assumed that insulin resistance is a key causative factor for diabetes and it has been demonstrated that individuals with peripheral insulin resistance are more prone to develop AD and related pathologies (Sims-Robinson et al., 2010; Rasgon et al., 2011). Moreover, at the cellular and molecular level, insulin signaling has been demonstrated to interfere with Aβ degradation and deposition (Carro et al., 2002; Farris et al., 2003). Further, insulin deficiency has also been shown to promote tau phosphorylation, leading to the accumulation of neurofibrillary tangles (Schubert et al., 2003). Accumulating evidence has shown that the brain itself develops insulin resistance due to the impairment in the insulin pathway (Moloney et al., 2010; Talbot and Wang, 2014; Su et al., 2017). In line with this, *in vivo* experimental data have shown that insulin resistance modifies cognitive performance even in the absence of diabetes (Su et al., 2017). Moreover, insulin signaling impairment has been found in brains from AD patients (Talbot et al., 2012). Increased levels of amyloid proteins have been found in the plasma of obese individuals (Lee et al., 2009; Jahangiri et al., 2013). Also, higher expression levels of beta-amyloid precursor protein and tau, two pathognomonic features of AD, have been found in the hippocampus of morbidly obese patients, compared to non-obese controls (Mrak, 2009; Nguyen et al., 2014). On the other hand, numerous studies have demonstrated that high-fat diets contribute to the higher expression of AD markers in rodents (Studzinski et al., 2009; Puig et al., 2012; Koga et al., 2014). Indeed, uncontrolled diabetes has also been linked to the risk of developing AD (Xu et al., 2009).

### Effects of Obesity-Associated Inflammation Within the Brain

Several aspects of brain function result affected by obesity-triggered inflammation. Periodic neuroinflammation is a necessary defense for the brain. However, when neuroinflammation becomes prolonged or uncontrolled (chronic neuroinflammation), it disrupts the normal protective barriers and leads to maladaptive synaptic plasticity and the development of different neurodegenerative disorders (Purkayastha and Cai, 2013). It has long been accepted that the BBB keeps blood inflammatory cells (monocytes and neutrophils) from getting into the brain. Therefore, microglia would be the only cells that mediate brain inflammation. However, it has become known that neutrophils and monocytes are able to infiltrate the injured brain and contribute to inflammation (Jeong et al., 2013). Astrocytes are known to produce anti-inflammatory factors that recruit monocytes, and neurons are able to both positively or negatively modulate anti-inflammatory response (Kim et al., 2010; Jeong et al., 2013). Thus, brain inflammation involves the coordinated efforts of several types of cells including microglia neutrophils, monocytes, astrocytes, and neurons.

Chronic neuroinflammation has been shown to impair adult hippocampal neurogenesis, and the blockade of neuroinflammation has demonstrated to restore it (Ekdahl et al., 2003; Monje et al., 2003). Also, impaired neurogenesis has been found in the hypothalamus of high-fat diet-fed rodents, probably due to the chronic neuroinflammatory response (Li et al., 2012). The complete mechanism is not fully understood, but stimulation of immune cells with the concomitant activation of the NF-kB pathway, and the release of interleukins and nitric oxide are thought to be involved (Purkayastha and Cai, 2013).

On the other hand, brain inflammation, mediated by inflammatory cells such as microglia and astrocytes, plays pivotal roles in regulating synaptic structure and function (Mottahedin et al., 2017). Synaptic disorganization is an integral part of several neurological disorders (Ebrahimi-Fakhari and Sahin, 2015). Glial cells are thought to play a vital role in synaptic architecture and hence neuronal connectivity. For this reason, factors that affect glial cells during development may also have long-term consequences on the synapse performance. Accordingly, an interaction between synaptic disorganization and immune function has been linked with cognitive weakness (Delpech et al., 2015). The BBB is known to actively participate in the inflammatory events and, conversely, the obesity-associated chronic inflammation also influences the BBB. It has been suggested that the BBB disruption occurs well before the infiltration of immune cells to the site of inflammation. Once within the brain, these effector cells and their secreted factors act upon microglia, astrocytes, and pericytes, which are important components of the BBB, and collaborate to a further BBB disruption thus leading to neuronal damage (Sonar and Lal, 2018).

Chronic brain inflammation also has been linked to neurodegenerative disorders such as AD. Aβ peptide accumulation in the brain parenchyma and blood vessels has been shown to promote both acute and chronic inflammatory responses which are mediated by astrocytes and microglia and which may finally cause neurodegeneration. However, the role of inflammation in AD is controversial, because inflammation has been found to have a beneficial role in the early stages of the disease. Nevertheless, the chronic activation of the microglia has been linked to the increased generation of Aβ and also with tau phosphorylation (Meraz-Ríos et al., 2013). Overall, the inflammatory process in AD is characterized by changes in microglial morphology together with astrogliosis (increased number, size, and motility of astrocytes). Studies in rodent models have shown that neuroinflammation is linked to early stages in tauopathies, even preceding tangle formation (Yoshiyama et al., 2007). Although the neuronal death associated with inflammation makes it a potential risk factor in the pathogenesis of AD, whether brain inflammation is the cause of or a secondary phenomenon to this disorder is unclear yet. Obesity may serve as an amplifier or initiator of the chronic inflammation observed in AD patients, although further research is needed to clarify the specific contribution of obesity to the chronic brain inflammation observed at the onset and progression of AD.

## Concluding Remarks

The causes and impact of obesity on overall health are far from linear and point to a complex set of interactions. The ultimate impact of obesity on an individual appears to be the summation of the effects of adipose-derived factors (adipokines, triglycerides, etc.) and indirect obesity effects (hypertension, glycemic dysregulation, etc.) and the physiology of the various organ systems of the body. Environmental factors and aging can accelerate or inhibit the effects of obesity on the various organ systems and tissues of the body, and this is an area of research that is rapidly expanding and identifying exciting results. Given the rapid increase in both obesity and aging in the populations of most Western societies, it will be critical to move obesity research into the realm of translational interventions, whereby the negative impacts of obesity on health are delayed or prevented in an increasingly elderly population.

## Author Contributions

RU and JK contributed to conception and design of the review and wrote the manuscript.

## Conflict of Interest Statement

The authors declare that the research was conducted in the absence of any commercial or financial relationships that could be construed as a potential conflict of interest.
